# Cytoplasmic TRAF4 contributes to the activation of p70s6k signaling pathway in breast cancer

**DOI:** 10.18632/oncotarget.2977

**Published:** 2015-01-09

**Authors:** Hua-Yan Ren, Jian Wang, Fan Yang, Xiao-Li Zhang, Ai-Lian Wang, Li-Li Sun, Ke-Xin Diao, En-Hua Wang, Xiao-Yi Mi

**Affiliations:** ^1^ From the Department of Pathology, First Affiliated Hospital and College of Basic Medical Sciences, China Medical University, Shenyang 110001, P.R. China

**Keywords:** TRAF4, p70s6k, TRAF2, cell proliferation, breast cancer

## Abstract

Tumor necrosis factor receptor associated factor 4 (TRAF4) is an important adaptor protein that plays a significant role in several signaling pathways. By studying the relationship between TRAF4 and 70 kDa ribosomal protein S6 kinase (p70s6k) *in vivo*, we demonstrated that cytoplasmic TRAF4 was correlated with the activation of p70s6k in breast cancer. Moreover, we found that cytoplasmic TRAF4 expression in breast cancer patients was significantly associated with a poor prognosis. To determine the exact mechanism, we analyzed the interaction between TRAF4 and p70s6k and identified the Zinc fingers domain of TRAF4 was responsible for their interaction in MCF7 cells. Furthermore, we found that activation of p70s6k/S6 signaling pathway by TRAF4 requires the mammalian target of rapamycin (mTOR) activity; TRAF4 acted as a sensitizer. Tumor necrosis factor receptor associated factor 2 (TRAF2), as a binding partner of TRAF4, could also promoted activation of p70s6k signaling via upregulating cytoplasm expression of TRAF4 and played a critical role in TNFa-induced activation of p70s6k/S6 pathway. Finally, we demonstrated p70s6k/S6 signaling pathway played an important role in the promoting function of TRAF4 on cell proliferation. In summary, our work suggests a new direction for understanding the oncogenic function of TRAF4 in breast cancer.

## INTRODUCTION

TRAF4 is one of seven tumor necrosis factor receptor associated factors (TRAFs). TRAFs share a common structural feature at the C terminus called TRAF domain. The family members are adaptor/scaffold proteins which can couple TNF receptors and interleukin receptors to the downstream signaling pathways. However, TRAF4 is a unique member of the family because it does not bind to TNF receptors and only weakly interacts with a few other receptors under certain conditions [1]. TRAF4 was initially identified in metastatic breast cancer [2]. It is overexpressed in several human cancers, including breast cancer [3]. Multiple subcellular localizations of TRAF4, including cytoplasmic, nuclear, and membrane have been described in other literature [2, 4, 5]. It is interesting that the localization of TRAF4 is altered in cancers. The biological implication of these multiple localizations however is not clear. Recently, one report revealed the significance of the nuclear localization of TRAF4 in breast cancers [6]. This study indicated that the nuclear localization of TRAF4 was associated with a poor survival rate in breast cancer patients following adjuvant chemotherapy. To date, the function of TRAF4 in the cytoplasm remains unclear. Our previous study showed that the cytoplasmic localization of TRAF4 is positively correlated with poorer cell differentiation in the tumor [7]. But the molecular mechanism is required for further research.

p70S6k (70 kDa ribosomal protein S6 kinase) is a serine/threonine kinase that is located downstream of the PI3K/AKT/mTOR (phosphoinosmde-3-kinase/protein kinase B/mammalian target of rapamycin) pathway. Numerous mitogens such as growth factors and hormones can activate the pathway to control cell growth, survival and cell cycle progression [8]. The activation of p70s6k is controlled by multiple phosphorylation events. The phosphorylation of the threonine 389 located in the Link domain is critical for activation of p70s6k *in vivo* [9]. Currently, mTOR is the most clear kinase that activates p70s6k by phosphorylating threonine 389 [10]. The activated p70s6k then phosphorylates 40S ribosomal protein S6, leading to an increased rate of translation of the class of 5′TOP mRNA transcripts. These transcripts are responsible for encoding critical components of the cellular translational machinery, thus accelerating protein synthesis. The mTOR/p70s6k signaling pathway is frequently activated in various types of human cancers [11, 12, 13, 14].

It has been reported that TRAF4 controls TNF induced S6 phosphorylation most likely by interacting with p70s6k [15]. However, the biological coupling between TRAF4 and p70s6k has not been firmly established by *in vivo* studies. Furthermore, the binding region of TRAF4 for p70s6k is also unknown. It is known that, unlike other TRAF family members, TRAF4 can not interact directly with TNF receptors. It is likely the other mechanism is responsible for transducing TNFa's signal intracellularly to TRAF4 and then to the p70s6k/S6 pathway. Our previous study identified the interaction between TRAF4 and TRAF2 [16]. TRAF2 can promote the survival and proliferation of cancer cells through NF-kB pathway by interacting with various TNF receptors. We hypothesize that TRAF2 mediates TNFa signaling that, in conjuction with TRAF4, leads to activation of the p70s6k pathway.

In this study, we show for the first time that the cytoplasm expression of TRAF4 in human breast-carcinoma was significantly associated with p70s6k activation and poor patient survival. Moreover, We provided evidence for the molecular mechanism of interaction between TRAF4 and p70s6k in MCF7 cells. In the study we also demonstrated TRAF2 was presumably the adaptor that mediated TNF receptors to TRAF4 and then p70s6k/S6 pathway. In the end, we demonstrated that the promoting effect of TRAF4 on the p70s6k/S6 pathway played a key role in its enhancing cell proliferation.

## RESULTS

### 1. TRAF4 cytoplasmic expression correlates with activation of p70s6k in breast cancer patients

#### 1.1 Relationship of TRAF4 and p-p70s6k (T389) expression in breast cancer

**Figure 1:**
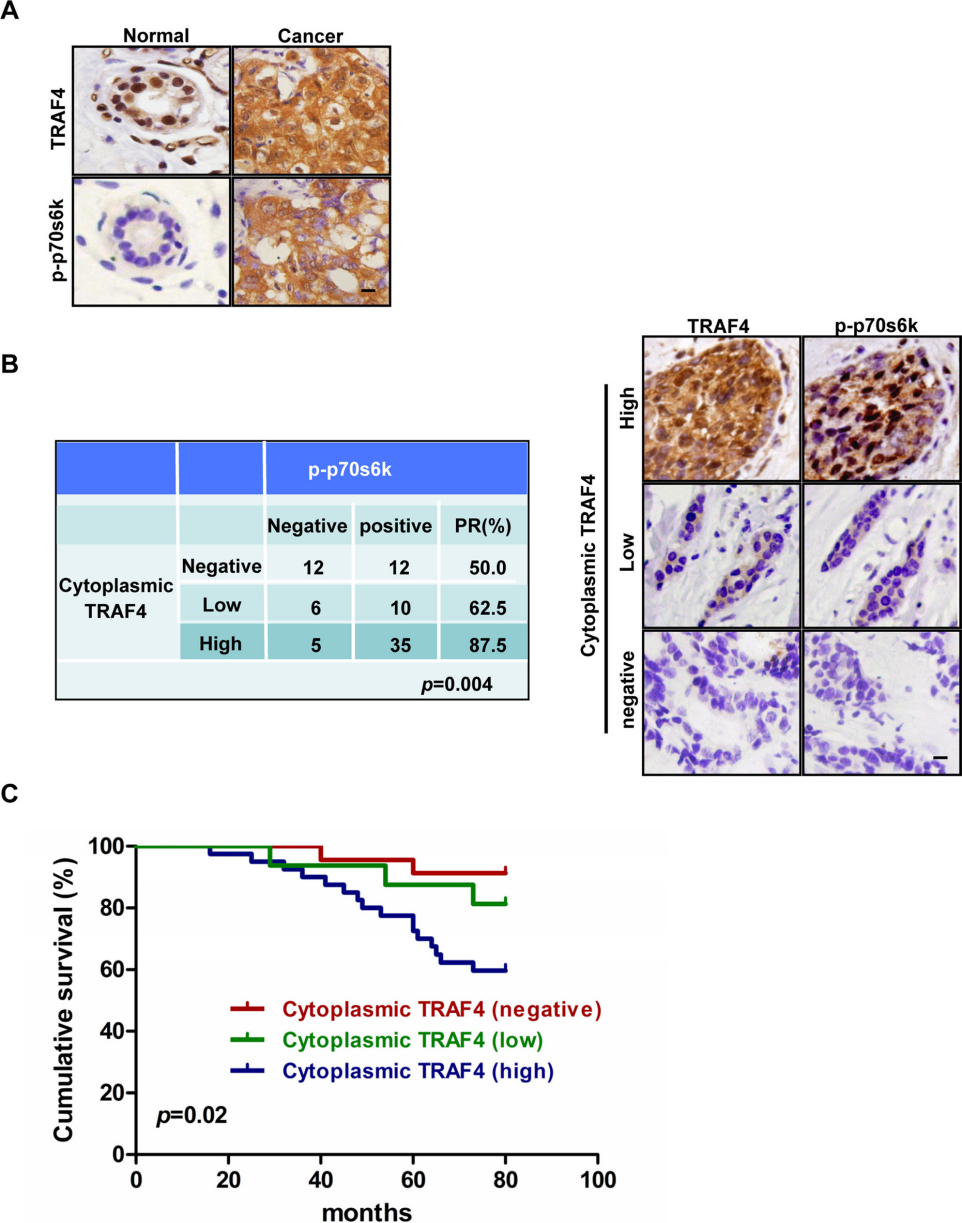
TRAF4 cytoplasmic expression correlates with activation of p70s6k and poor survival in breast cancer patients **(A)** Immunohistochemical analysis of TRAF4 and p-p70s6k(T389) localization in normal breast tissue (NBT) and breast cancer tissue (BCT). Bar, 20 μm. **(B)** The positive rate of p-p70s6k expression was positively correlated with cytoplasmic TRAF4 expression level in breast cancer patients (chi-square test, *p* = 0.004). Bar, 10 μm. **(C)** Kaplan-Meier analysis of overall survival curves of cytoplasmic TRAF4 expressions in 80 cases of breast cancer patients was shown. High Cytoplasm expression of TRAF4 significantly correlated with patient poor survival (log-rank test, *p* = 0.02).

In order to identify whether the expression of TRAF4 correlates with the activation of p70s6k *in vivo*, we detected the expression of TRAF4 and p-p70s6k (T389) in normal breast tissue(NBT) (*n* = 16) and breast cancer tissue (BCT) (*n* = 80). As shown in Figure 1A, TRAF4 was predominantly positively immunostained in the nucleus of normal breast epithelial cells, while besides the nuclear stain, the cytoplasm expression of TRAF4 was obviously found in breast cancer cells. Statistically, cytoplasmic TRAF4 was highly expressed in BCT (56 out of 80, 70.0%), compared with NBT (5 out of 16, 31.3%) (*p* < 0.05) (Table 1). This indicated the localization of TRAF4 in the cytoplasm may contribute to the progression of breast cancer. p-p70s6k was negative in NBT, which was consistent with other study [17], while the immunostain of p-p70s6k was positive in BCT (Figure 1A). In order to confirm whether cytoplasmic TRAF4 expression was correlated with the phosphorylation and activation of p70s6k. the association of cytoplasmic TRAF4 expression with p-p70s6k expression in BCT was analyzed. As shown in Figure 1B, we obviously found that the high expression group of cytoplasmic TRAF4 has much higher positive rate of p-p70s6k comparing with negative and low groups (*p* < 0.05, chi-square test). p-p70s6k expression was positively correlated with cytoplasmic TRAF4 expression.

**Table 1:** Cytoplasmic TRAF4 and p-p70s6k expression in breast carcinogenesis

Groups	N	Cytoplasmic TRAF4		PR(%)	*p*-p70s6k		PR(%)
		negative	positive		negative	positive	
NBT	16	11	5	31.3	16	0	00.0
BCT	80	24	56	70.0*	23	57	71.3*

NBT, normal breast tissue.

BCT, breast cancer tissue.

PR, positive rate; * compared with NBT, Pearson Chi-Square (Cytoplasmic TRAF4) & Fisher's Exact Test (p-p70s6k), *p* < 0.05.

#### 1.2 Prognostic values of the cytoplasmic expression of TRAF4

It has been reported that p-p70s6k was related to worse prognosis in breast cancer [17]. We found that cytoplasm expression of TRAF4 was associated with p-p70s6k in the study. To examine whether cytoplasmic TRAF4 expression has clinical significance, the breast cancer patients were followed up with for 80 months. We analyzed the relationship between TRAF4 with the overall survival of these breast cancer patients after surgery was performed by the Kaplan-Meier method. Although it is reported that high TRAF4 nuclear expression is also associated with poor survival in breast cancer patients [6], High cytoplasm expression of TRAF4 was significantly correlated with poor survival of breast cancer patients (log-rank test, *p* = 0.02), as shown in Figure 1C.

#### 1.3 P70s6k is highly activated in breast cancer tissue sample and breast cancer cells which had high TRAF4 expression

**Figure 2:**
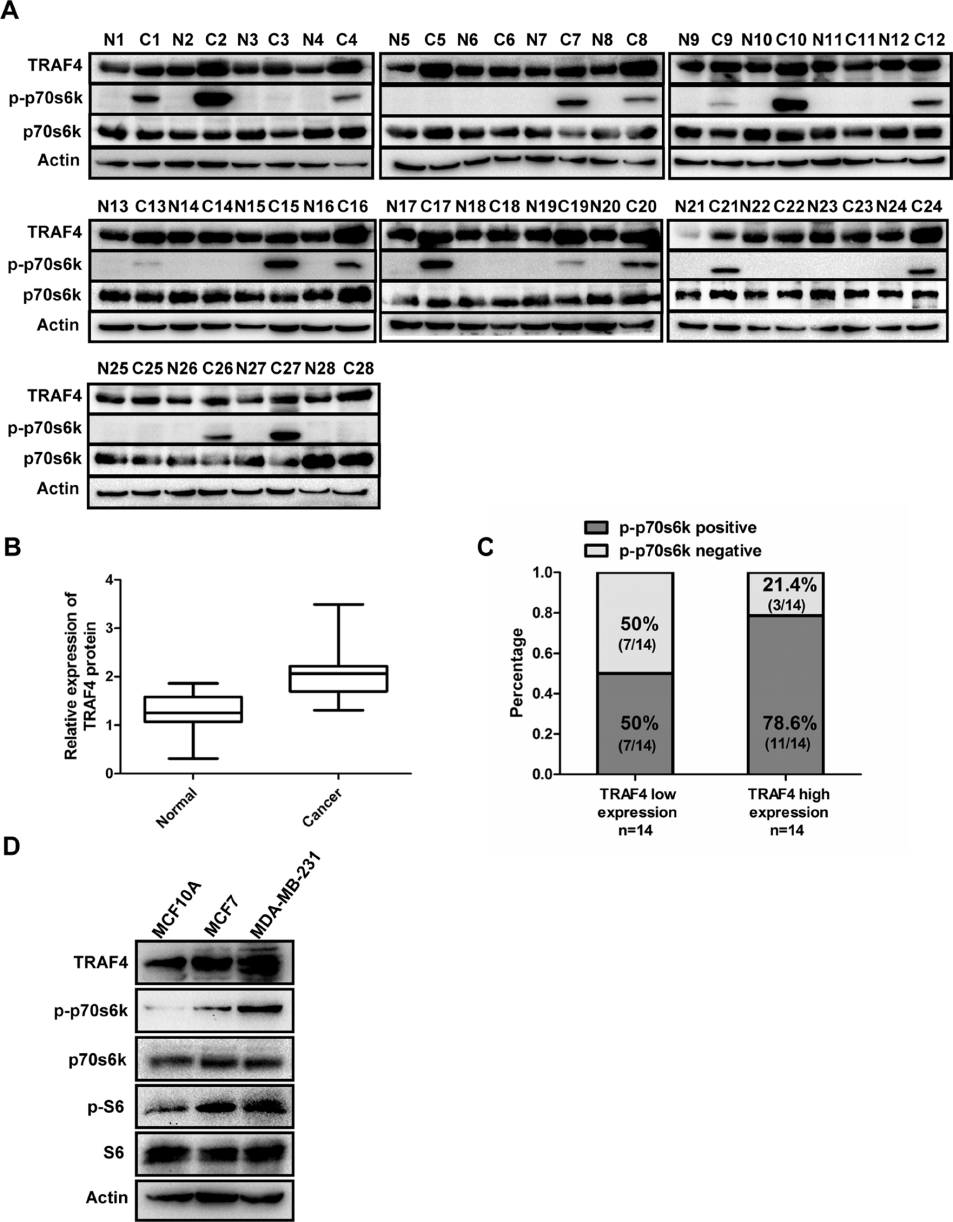
Expression of TRAF4 is correlated with p-p70s6k in breast cancer tissues and breast cancer cells **(A)** Expression of TRAF4, p-p70s6k (T389) and p70s6k detected by Western Blot in 28 breast cancer tissues and paired normal tissues. Actin was used as an internal control. **(B)** Boxplot indicated TRAF4 expression in breast cancer and paired normal tissues. The intensity of protein bands were measured, the y-axis of boxplot was based on the relative expression of TRAF4 to Actin. The expression of TRAF4 was higher (mean = 2.088) in breast cancer tissues than that in paired normal tissues (mean = 1.285) (Student's *t* test, *p* < 0.05). **(C)** Positive rate of p-p70s6k expression was shown grouped by the mean relative (min = 1.31, max = 3.49, mean = 2.088) expression level of TRAF4 in 28 cancerous tissue samples. The positive rate of p-p70s6k in TRAF4 high expressers was higher (11 out of 14, 78.6%) than that in TRAF4 low expressers (7 out of 14, 50%) (Fisher's Exact Test, *p* < 0.05). **(D)** TRAF4 and p-p70s6k/p-S6 expression levels in non-malignant breast epithelial cell line MCF-10A and breast cancer cell lines MCF-7 and MDA-MB-231 were analyzed by Western Blot. TRAF4, p-p70s6k and p-S6 expression was higher in the MCF7 and MDA-MB-231 cells than that in the MCF10A cells.

To better understand the correlation between TRAF4 and p-p70s6k, the expression of TRAF4, p70s6k and the phosphorylation status of p70s6k were further examined in 28 cases of freshly paired breast cancer tissues. As demonstrated in Figure 2A, 2B, dramatically elevated TRAF4 was observed in breast cancer tissues than in paired normal breast tissue (*p* < 0.05, Student's *t* test). p-p70s6k had a positive expression in 18 out of 28 cancer samples, and was undetectable in paired normal breast tissues (Figure 2A), which was consistent with the immunohistochemistry results (Figure 1A). According to relative expression level of TRAF4 to Actin, we divided the breast cancer samples equally into two groups, TRAF4 low expressers and high expressers. As shown in Figure 2C, tumors with high expression of TRAF4 revealed a higher positive rate of p-p70s6k (11 out of 14, 78.6%) than the low group (7 out of 14, 50.0%) (Fisher's Exact Test, *p* < 0.05). These results indicate a positive correlation between TRAF4 and p-p70s6k expression in tissue samples. In addition, we detected the expression of TRAF4 and p-p70s6k/p-S6 in breast cancer cells MCF7 and MDA-MB-231 along with non-malignant breast epithelial cells MCF10A by Western blotting. As shown in Figure 2D, p-p70s6k was nearly undetectable in MCF10A, but there was obvious expression of p-p70s6k in MCF7 and MDA-MB-231. The expression pattern of TRAF4, p-p70s6k, and p-S6 were almost the same as each other from MCF10A to MCF7 and MDA-MB-231, which indicated that TRAF4 may be involved in the activation of p70s6k/S6 pathway.

### 2. TRAF4 interactes with p70s6k through Zinc fingers domain

**Figure 3:**
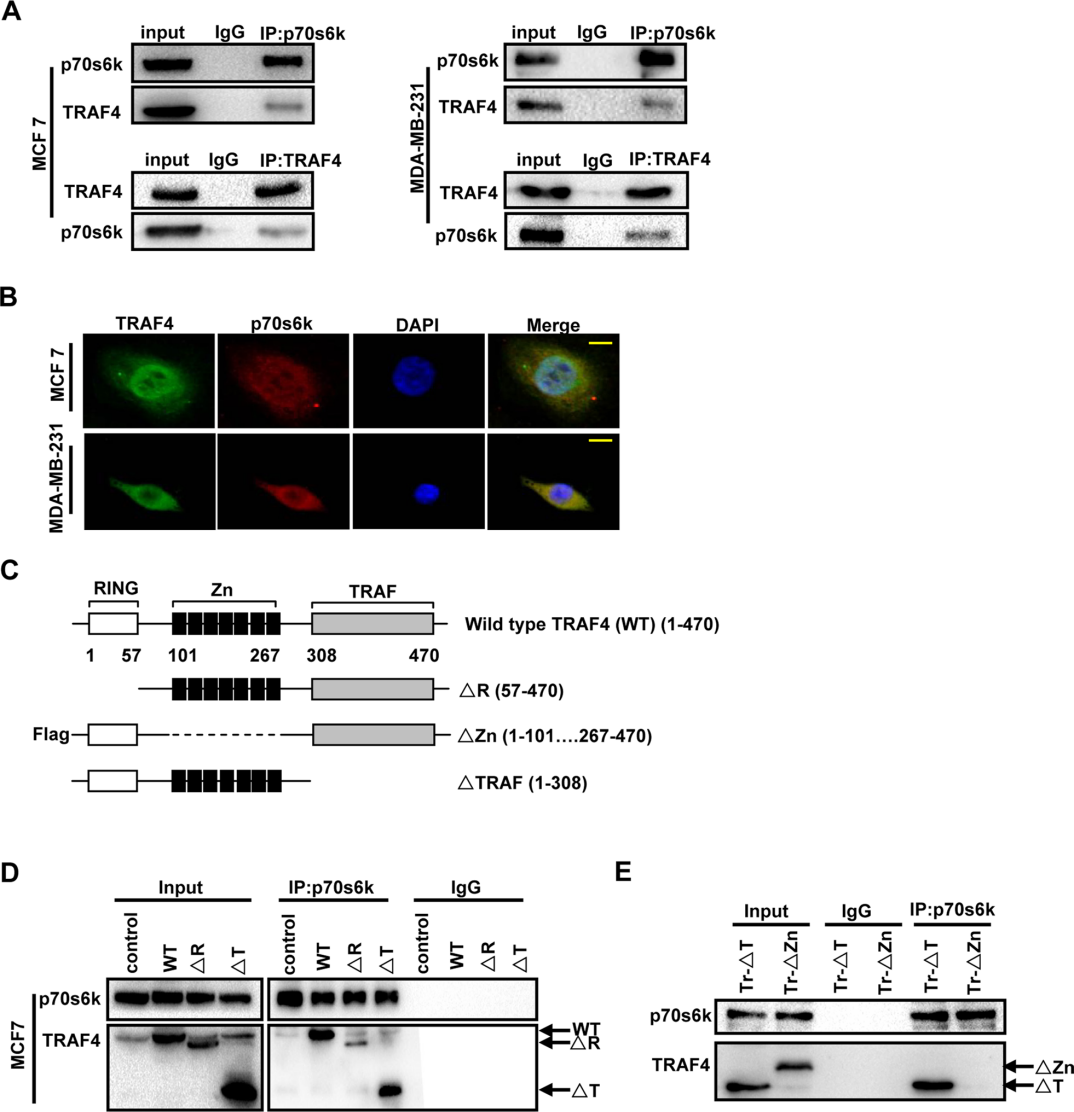
TARF4 interacts with p70s6k through Zinc fingers domain **(A)** TRAF4 could interact with p70s6k each other in breast cancer cell lines MCF7 and MDA-MB-231. TRAF4 and p70s6k were dragged down by co-immunoprecipitation using p70s6k or TRAF4 antibodys respectively followed by Western blot analysis both in MCF7 and MDA-MB-231. **(B)** Co-localization of TRAF4 and p70s6k in MCF7 and MDA-MB-231 by immunofluorescence analysis. TRAF4 (green), p70s6k (red), bar, 10 μm. **(C)** Schematic representations of TRAF4 wild type (WT, 1–470), the Ring domain-deleted (ΔR, 57–470), Zinc fingers domain-deleted (ΔZn, 1–101 267–470) and TRAF domain-deleted (ΔT, 1–308) mutants. **(D)** The Ring finger domain and TRAF domain are indispensable for TRAF4 binding to p70s6k in MCF7. The plasmids for Wild type TRAF4, ΔR and ΔT mutants were transfected into MCF7 cells. The cell lysates were then incubated with p70s6k antibody and protein A/G agarose beads. Western blot was performed to analysis the mutants that co-immunoprecipitation by p70s6k antibody. **(E)** The Zinc fingers domain is important for TRAF4 binding to p70s6k in MCF7. Zn Δ mutant was transfected into MCF7 cells, co-immunoprecipitation using p70s6k antibody and Western blot were performed as before. T mutant was used as a positive control.

TRAF4 interacted with p70s6k in the erythroleukemia cell line [15]. To confirm whether the interaction between TRAF4 and p70s6k only occurred in specific cells, we tested breast cancer cells MCF7 and MDA-MB-231. Consistent with the previous study, TRAF4 could interact with p70s6k both in MCF7 and MDA-MB-231 (Figure 3A). Moreover, we detected the localization of TRAF4 and p70s6k in MCF7 and MDA-MB-231 by immunofluorescence analysis. As shown in Figure 3B, we found co-localization of TRAF4 and p70s6k both in MCF7 and MDA-MB-231. These results indicate that the interaction between TRAF4 and p70s6k was not a cell specific event, but the p70s6k-interacting region in TRAF4 is still unknown. As shown in Figure 3C, TRAF4 contains a RING domain, seven Zinc finger motifs and a homologous TRAF domain [1, 18, 19]. The N-terminal RING finger motif has potential E3 ubiquitin ligase activity [20, 21, 22]. The TRAF domain is responsible for its interaction with several receptor family members [23]. To analyze the regions important for TRAF4 interacting with p70s6k, the expression vectors for wild type TRAF4, RING, Flag-tagged Zinc fingers, or TRAF domain-deleted mutants were transfected into MCF7. A co-immunoprecipitation experiment was then performed using p70s6k and Flag tag antibodies along with an IgG as control. The RING domain and TRAF domain were dispensable for the interaction, since ΔR and ΔTRAF can still be dragged down by p70s6k antibody (Figure 3D). The Zinc fingers domain deletion mutant (Flag-ΔZn) could not be dragged down by p70s6k antibody. (Figure 3E). These results suggested that Zinc fingers domain was required for TRAF4 interaction with p70s6k.

### 3. TRAF4 promotes the activation of p70s6k signaling through enhancing the interaction between mTOR and p70s6k

#### 3.1 TRAF4 promotes the activation of p70s6k signaling pathway

**Figure 4:**
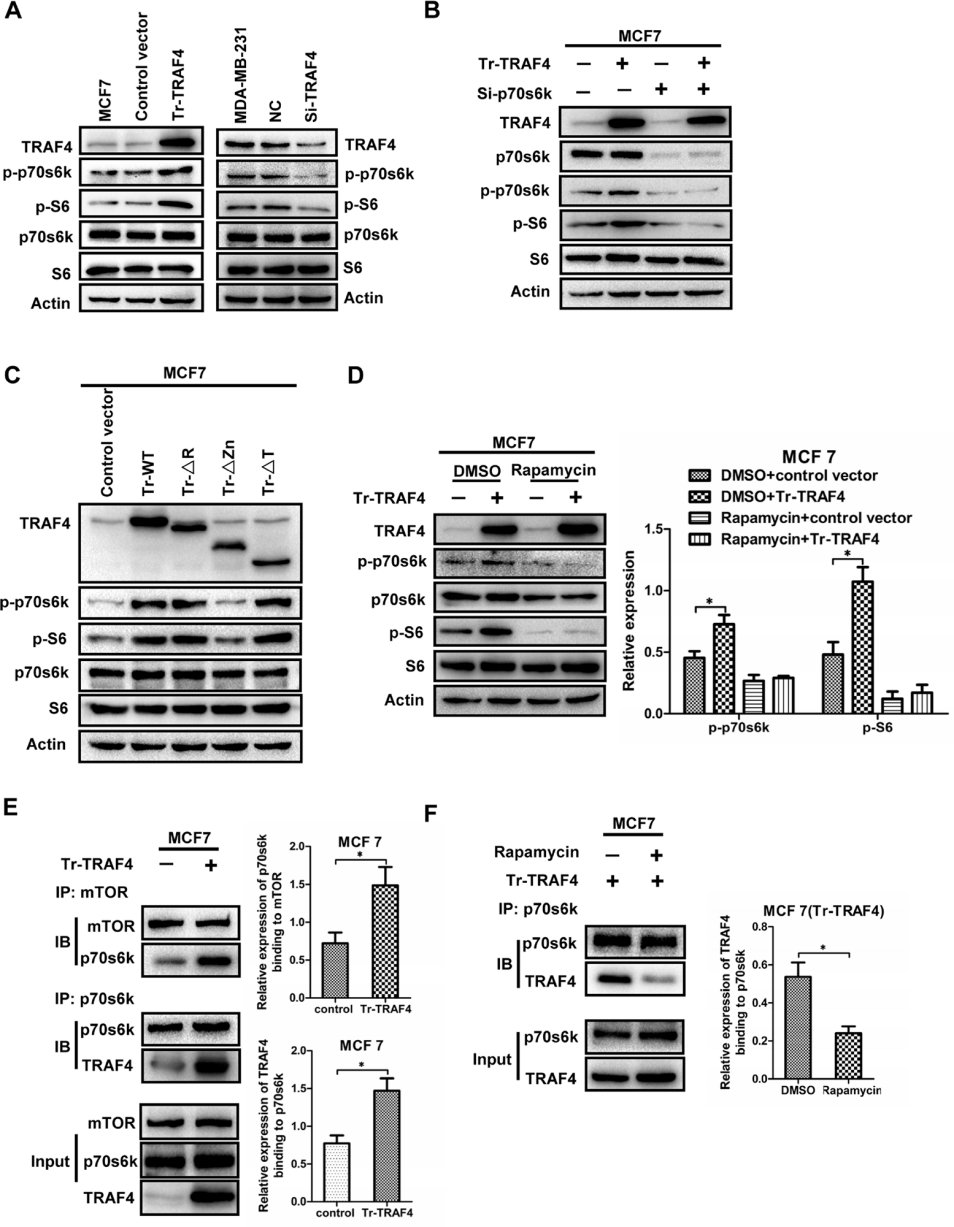
TRAF4 promotes the phosphorylation of p70s6k through enhancing the interaction between mTOR and p70s6k **(A)** TRAF4 promotes the phosphorylation of p70s6k and S6. The Western blot analysis indicated that overexpression of TRAF4 in MCF7 significantly increased the phosphorylation of p70s6k/S6 when compared with control vector. While, knocking down of TRAF4 in MDA-MB-231 significantly reduced phosphorylation level of p70s6k/S6 when compared with negative control. **(B)** TRAF4 promotes the phosphorylation of S6 through p70s6k. p-S6 expression significantly increased after TRAF4 plasmid was transfected into MCF7. while, elevation of p-S6 expression was obviously inhibited when TRAF4 expression vector together with p70s6k specific siRNA were transfected into MCF7. **(C)** Zinc fingers domain of TRAF4 is responsible for its promoting effect on p70s6k/S6 signaling. The protein levels of p-p70s6k and p-S6 were significantly increased after the expression vectors for wild type TRAF4(WT), ΔR, and ΔT mutants were transfected into MCF7 cells when compared with control empty vector. But, there were no obvious changes on p-p70s6k and p-S6 protein level after Flag-tagged-ΔZn mutant was transfected into MCF7 when compared with control empty vector. **(D)** mTOR activity is predominantly required for the promoting effect of TRAF4 on enhancing activation of p70s6k/S6 pathway. The elevation of p-p70s6k and p-S6 expression was obviously inhibited when TRAF4 overexpression together with Rapamycin treatment. The relative expression levels of protein were calculated in reference to Actin as the loading control. **(E)** TRAF4 promotes the interaction of p70s6k and mTOR. The relative expression of p70s6k binding to mTOR was calculated in reference to mTOR as the loading control. **(F)** Rapamycin inhibits the interaction between TRAF4 and p70s6k. The expression of TRAF4 that binding to p70s6k was siginificantly reduced in Rapamycin group than that in control group. The relative expression of TRAF4 binding to p70s6k was calculated as (E) Data was presented as mean ± SD from three independent experiments. (*p < 0.05).

The study showed that TRAF4 was correlated with p-p70s6k *in vivo*. However, the molecular mechanism of TRAF4's effect on p70s6k/S6 phosphorylation remains unknown. Due to the higher expression of TRAF4 than MCF7 in MDA-MB-231 (Figure 2D), we transfected full length TRAF4 vector into MCF7 and employed siRNA to knockdown TRAF4 expression in MDA-MB-231. We then detected the protein levels of p-p70s6k and p-S6. The results in Figure 4A showed that overexpression of TRAF4 in MCF7 significantly increased the phosphorylation of p70s6k and S6, and had no effect on total p70s6k and S6 protein expression. Meanwhile knockdown of TRAF4 in MDA-MB-231 significantly reduced phosphorylation of p70s6k and S6 (Figure 4A). As we know, S6 is a well-known substrate for p70s6k. But S6 can also be phosphorylated by other kinases such as p90S6 kinase (RSK), which is the substrate of ERK [24]. In order to test whether p70s6k is the mediator that helps TRAF4 promote the elevation of p-S6 expression, p70s6k was depleted by specific siRNA along with overexpression of TRAF4 in MCF7. As shown in Figure 4B, knockdown of p70s6k strongly decreased the level of p-S6 and abolished TRAF4-induced p-S6 up-regulation. These results suggested that p70s6k was required for TRAF4-mediated p-S6 regulation. In summary, TRAF4 promoted the activation of p70s6k/S6 signaling pathway.

#### 3.2 Zinc fingers domain of TRAF4 is required for its promoting effect on p70s6k/S6 pathway

To confirm which domain in TRAF4 was responsible for its promoting effect on p70s6k/S6 pathway, we transfected the expression vectors for wild-type TRAF4 and domain-deleted mutants into MCF7. As shown in Figure 4C, wild-type TRAF4, ΔR and ΔT mutants were all able to elevate the phosphorylation level of p70s6k and S6 except ΔZn mutant. These results indicated that the Zinc fingers domain was required for TRAF4 to promote p70s6k/S6 phosphorylation and activation. Moreover, because of the indispensable role of the Zinc fingers domain in the interaction between TRAF4 and p70s6k, it indicated that the interaction may be involved in the promoting function of TRAF4 on p70s6k/S6 phosphorylation.

#### 3.3 mTOR activity is predominantly required for the promoting effect of TRAF4 on enhancing activation of p70s6k/S6 pathway

mTOR is a serine/threonine kinase that lies upstream of p70s6k. It can phosphorylate and activate p70s6k. Rapamycin is an immunosuppressant that inhibits mTOR by binding to the phosphatidic acid-binding site required for mTOR activation [25, 26]. Because TRAF4 is not a protein kinase and it cannot phosphorylate p70s6k directly, mTOR may be involved in the promoting effect of TRAF4 on enhancing phosphorylation of p70s6k. To confirm this point, MCF7 cells were treated with mTOR inhibitor rapamycin. As shown in Figure 4D, after the treatments with rapamycin, significantly lower or absent protein expression of p-p70S6k and p-S6 was detected by Western blotting in MCF7 cells as compared to non-treated samples. Moreover, inhibition of mTOR activity by rapamycin significantly inhibited the promoting effect of TRAF4 on phosphorylation of p70s6k, Although there was still a seemingly weak upregulation of p-S6, it was not significant. This may be due to that Rapamycin can not completely inhibit the phosphorylation and activation of p70s6k by mTOR, which also can be explained by the presence of Rapamycin-insensitive mTOR (Figure 4D). These results indicate that the promoting effect of TRAF4 on activation of p70s6k/S6 pathway was predominantly dependent on mTOR activity.

#### 3.4 TRAF4 acts as a sensitizer on the interaction between mTOR and p70s6k

mTOR could phosphorylate and activate p70s6k through interacting with it [10]. Although TRAF4 promoted the p70s6k/S6 pathway through mTOR, the exact molecular mechanism was still unknown. To demonstrate the interaction among TRAF4, p70s6k and mTOR, we transfected the TRAF4 expression vector into MCF7 with or without Rapamycin treatment and then detected the binding capacity between mTOR with p70s6k and TRAF4 with p70s6k by co-immunoprecipitation and Western blotting. As shown in Figure 4E, overexpression of TRAF4 increased the interaction between p70s6k and mTOR, which indicated that more p70s6k proteins were phosphorylated and activated by mTOR. We also found that more TRAF4 proteins were going to interact with p70s6k after TRAF4 expression vector was transfected into MCF7 cells (Figure 4E). However, when TRAF4 vector was transfected into MCF7 together with rapamycin treatments, the protein level of TRAF4 that binded to p70s6k was significantly reduced (Figure 4F). These results suggest that TRAF4 overexpression enhanced the ability of mTOR to interact and phosphorylate p70s6k. Meanwhile, mTOR activity was also required for interaction of TRAF4 and p70s6k. In summary, TRAF4 acted as a sensitizer.

### 4. TRAF2 promotes p70s6k/S6 pathway partially through its regulation on cytoplasmic/nuclear distribution of TRAF4

#### 4.1 TRAF2 promotes p70s6k/S6 pathway and regulates the distribution of TRAF4

**Figure 5:**
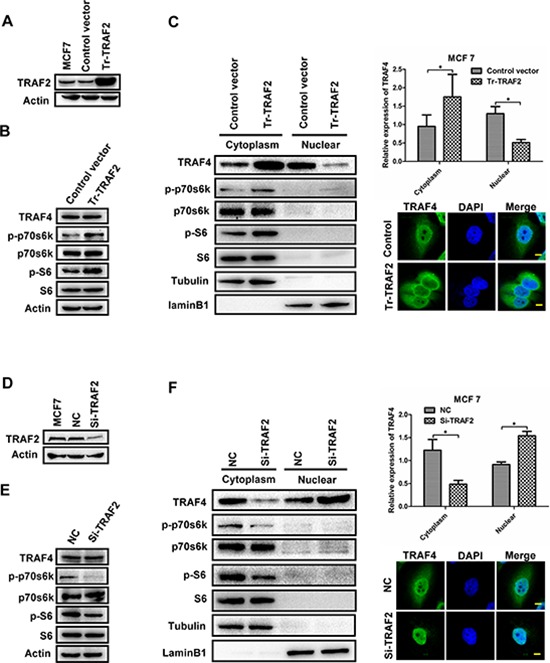
TRAF2 promotes p70s6k/S6 pathway and regulates the distribution of TRAF4 **(A)** Western blot of TRAF2 transfection efficiency in MCF7 cells. **(B)** Overexpression of TRAF2 in MCF7 cells significantly increased the protein level of p-p70s6k and p-S6. **(C)** TRAF2 overexpression upregulated the cytoplasmic TRAF4 and reduced nuclear TRAF4. Western blot was performed to detect the distribution of TRAF4 together with the immunofluorescence staining. Bar, 10 μm. **(D)** Western blot of TRAF2 depletion efficiency in MCF7 cells. **(E)** Knocking down of TRAF2 in MCF7 significantly reduced phosphorylation level of p70s6k/S6. **(F)** Knocking down TRAF2 reduced the cytoplasmic TRAF4 and increased nuclear TRAF4. Bar, 10 μm. The relative expression levels of protein were calculated in reference to Tubulin or laminB1 as the loading control. Tubulin was used as cytoplasm control. laminB1 was used as nuclear control. Data was presented as mean ± SD from three independent experiments. (*p < 0.05).

The phosphorylation and activation of p70s6k predominantly occurred in the cytoplasm of cells, therefore the promoting effect of TRAF4 on p70s6k/S6 pathway most likely depended on the cytoplasmic TRAF4. Our previous study had confirmed that TRAF2 could regulate the distribution of TRAF4, such as overexpression of TRAF2 increasing the cytoplasmic TRAF4 in MCF7 [16], so we suspected that TRAF2 could affect the phosphorylation of p70s6k and S6 and that TRAF4 may be involved in this process. To confirm our suspicion, we employed TRAF2 expression vector and TRAF2 specific siRNA into MCF7 cells; Western blotting was used for analysis. As shown in Figure 5A, 5B, 5C, up-regulation of TRAF2 significantly increased the phosphorylation level of p70s6k and S6 in MCF7; meanwhile, overexpression of TRAF2 increased the cytoplasmic TRAF4 and reduced nuclear TRAF4 without affecting the total TRAF4 expression, which was also shown by immunofluorescence staining. Down-regulation of TRAF2 notably reduced the protein level of p-p70s6k (T389) and p-S6 (S235/236), TRAF2 knockdown significantly reduced the cytoplasmic TRAF4 and increased nuclear TRAF4 without any change in total TRAF4 expression (Figure 5D, 5E, 5F). These results suggested that TRAF2 could regulate the distribution of TRAF4, which was consistent with our previous study [16]. Moreover, TRAF2 promoted the p70s6k/S6 pathway.

#### 4.2 TRAF2 promotes the phophorylation of p70s6k and S6 through TRAF4

**Figure 6:**
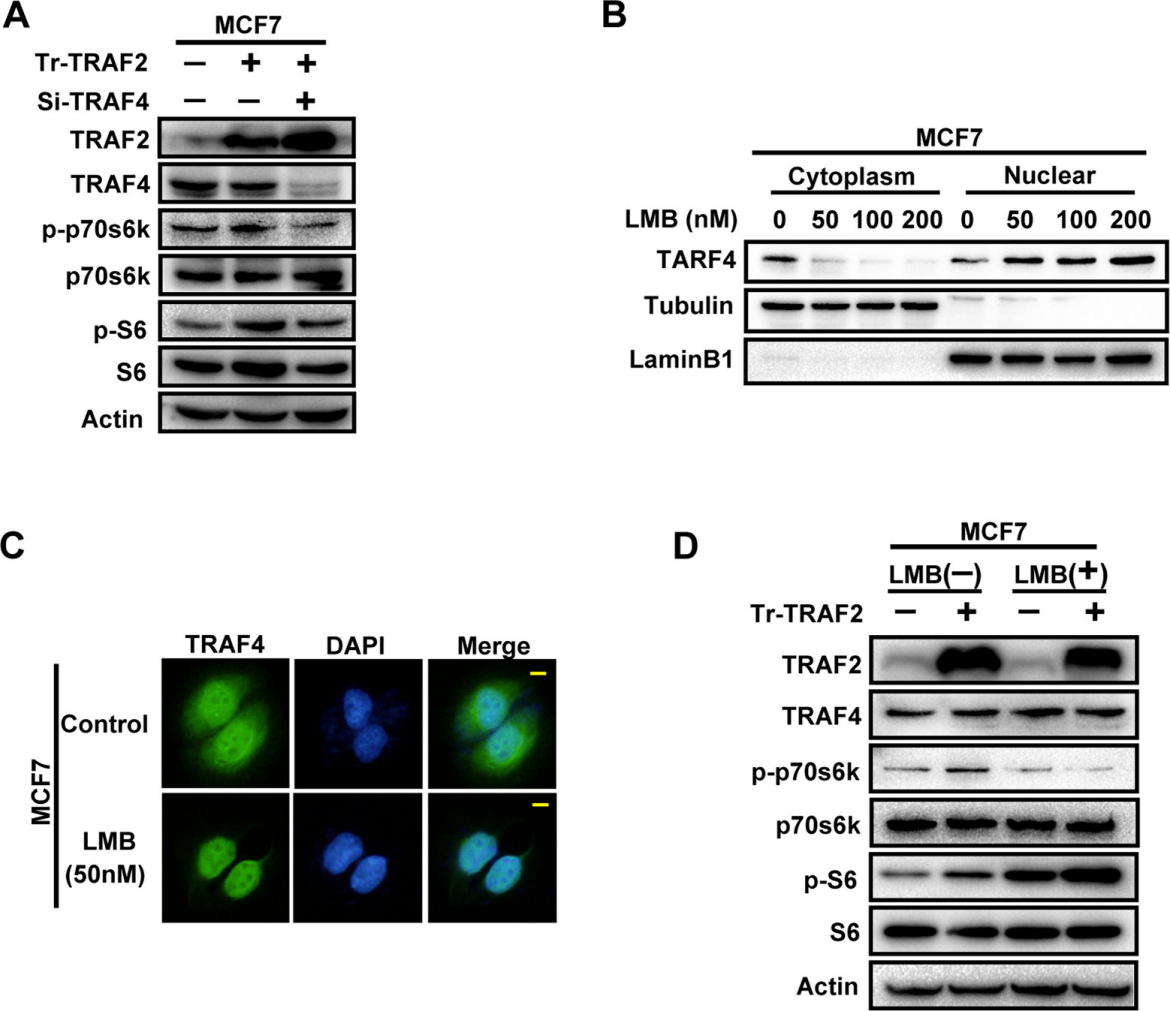
The promoting effect of TRAF2 on p70s6k/S6 pathway is partially through its regulation on cytoplasmic/nuclear distribution of TRAF4 **(A)** TRAF2 promoted the phosphorylation of p70s6k and S6 through TRAF4. Koncking down TRAF4 significantly abolished TRAF2-induced p-p70s6k and p-S6 up-regulation. **(B)** The inhibition efficiency of LMB to nuclear export of TRAF4 at different concentration in MCF7 cells. MCF7 cells were treated with LMB at 50 nM, 100 nM, and 200 nM for 20 h, and then nuclear/cytoplasmic extraction and Western blot were performed to detected the inhibition efficiency of LMB. **(C)** Immunofluorescence analysis of the inhibition status of LMB to nuclear export of TRAF4 at 50 nM in MCF7 cells. 50 nM LMB could significantly inhibite the nuclear export of TRAF4 and keep most of it in the nuclear. TRAF4 (green), bar, 10 μm. **(D)** The promoting effect of TRAF2 on p70s6k phosphorylation was significantly suppressed by TRAF4 nuclear retention induced by LMB. TRAF2 expression vector and control vector were transfected into MCF7 cells with or without LMB treatment and western blot was performed to detect p-p70s6k protein level. LMB which inhibited nuclear exportion of TRAF4 significantly abolished the function of TRAF2 promoting p70s6k phosphorylation.

In order to assess whether TRAF4 was a key component in the increased function of TRAF2 on p70s6k/S6 pathway, we employed the TRAF2 expression plasmid into MCF7 together with TRAF4 specific siRNA to knockdown TRAF4 expression. Compared to MCF7 cells transfected with TRAF2 vector alone, MCF7 cells that expressed TRAF2 plasmid and siRNA-TRAF4 significantly abolished TRAF2-induced p-p70s6k and p-S6 up-regulation, as shown in Figure 6A. These results suggest that TRAF4 is involved in promoting TRAF2 in the p70s6k/S6 pathway. In other words, TRAF2 promoted p70s6k signaling through TRAF4.

#### 4.3 The promoting effect of TRAF2 on p70s6k/S6 pathway is at least partially through its regulation on cytoplasmic/nuclear distribution of TRAF4

To test whether TRAF2-induced up-regulation of p-p70s6k and p-S6 was a result of its regulation on distribution of TRAF4, we employed Leptomycin B(LMB) into MCF7 to inhibit nuclear export of TRAF4. We first evaluated the cytoplasmic/nuclear distribution status of TRAF4 after LMB treatments by Cellular fractionation and western blotting and then determined the most effective working concentration of LMB. As shown in Figure 6B, 50 nM, 100 nM and 200 nM all could inhibit the nuclear export of TRAF4 effectively after a 20 hour incubation. To reduce the toxicity of LMB to the cells, we chose 50 nM as the working concentration as it had been shown to cause significant inhibition by immunofluorescence staining (Figure 6C). Next, we transfected TRAF2 expression vector into MCF7 cells, which then were incubated with LMB to inhibit nuclear export of TRAF4. As shown in Figure 6D, when MCF7 cells were transfected with TRAF2 plasmid, and incubated with LMB to inhibit nuclear TRAF4 export, the promoting effect of TRAF2 on p70s6k phosphorylation was significantly reduced. Surprisingly, the promoting effect of TRAF2 on S6 phosphorylation was not abolished, as we could still find the elevation of p-S6 protein level after TRAF2 overexpression together with LMB treatment. LMB itself could also increase the phosphorylation level of S6, which may be attributed to the unspecific inhibition of LMB to TRAF4 nuclear exportation. These results suggest that the promoting effect of TRAF2 on the p70s6k/S6 pathway was at least partially through its regulation on cytoplasmic/nuclear distribution of TRAF4.

### 5. TRAF2 mediates the phosphorylation of p70s6k induced by TNFa, which is dependent on the participation of TRAF4

**Figure 7:**
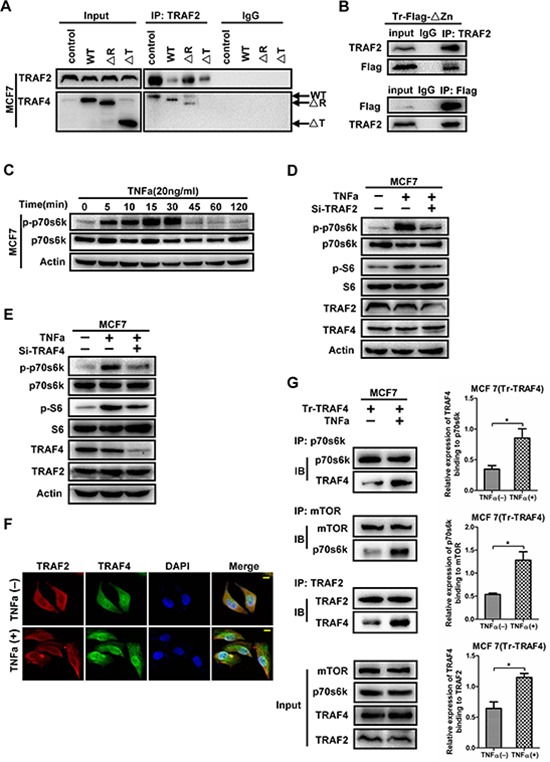
TRAF2 mediates the phosphorylation of p70s6k induced by TNFa, with the participation of TRAF4 **(A)** The TRAF domain is required for TRAF4 interacting with TRAF2. Wild type, ΔR and ΔT mutants were transfected into MCF7 cells. The cell lysates were then incubated with TRAF2 antibody and protein A/G agarose beads. Western blot was performed to analysis the mutants that co-immunoprecipitation by TRAF2 antibody. **(B)** The Zinc fingers domain is indispensable for TRAF4 binding to TRAF2 in MCF7. Flag tagged ΔZn mutant was transfected into MCF7 cells, co-immunoprecipitation using TRAF2 or Flag antibody and Western blot were performed as before. Both TRAF2 and Flag could be dragged down by each other. **(C)** TNFa induces phosphorylation and activation of p70s6k. MCF7 cells were treated with TNFa (20ng/ml) for different time. Western blot was to detect p-p70s6k protein level. The phosphorylation of p70s6k peaked at 15minutes and maintained until 30 minutes. **(D, E)** Both TRAF4 and TRAF2 play critical role in the activation of p70s6k/S6 pathway induced by TNFa. Koncking down TRAF2 or TRAF4 significantly reduced the activation of p70s6k/S6 pathway induced by TNFa. **(F)** TRAF2 lies upstream of TRAF4 in TNFa-induced p70s6k pathway activation. The distribution of TRAF2 and TRAF4 after TNFa treatment were detected by immunofluorrescence staining. TRAF2 had obviously distribution of cytosolic side near the cell membrane, and co-localization of TRAF4 and TRAF2 was obviously found in this section after TNFa stimulation. TRAF2 (red), TRAF4 (green), bar, 10 μm. **(G)** The interactions between mTOR and p70s6k, p70s6k and TRAF2, TRAF2 and TRAF4 were enhanced after TNFa treatment. TRAF4 expression vector was employed into MCF7 together with or without TNFa. The interaction between them were detected by co-immunoprecipitation and Western blotting. After TNFa stimulation, more p70s6k interacted with mTOR, more TRAF4 interacted with p70s6k, more TRAF4 interacted with TRAF2. The relative expression levels of TRAF4 binding to p70s6k, p70s6k binding to mTOR, TRAF4 binding to TRAF2 were calculated as before. Data was presented as mean ± SD from three independent experiments. (*p < 0.05).

Our previous study has found the interaction between TRAF2 and TRAF4 [16], however, which TRAF2-interacting region in TRAF4 remains unknown. To analyze the regions relevant to TRAF4 interacting with TRAF2, A co-immunoprecipitation experiment was then performed as before. As shown in Figure 7A and 7B, the TRAF domain was required for TRAF4 interacting with TRAF2 since ΔTRAF was not dragged down by TRAF2 antibody, which is consistent with the interactions between other TRAF members [27]. TRAF4 was involved in p-S6 upregulation induced by TNFa [15], but which protein transfer signals from TNFR to TRAF4 is still unknown. To confirm whether TRAF2 was the adaptor, we first evaluated the phosphorylation status of p70s6k and determined the most effective working time of TNFa. As shown in Figure 7C, the level of p-p70s6k peaked at the fifteenth minute and maintained peak level until 30 minutes, so we adopted 20 minutes in our following operation. Next, we employed TRAF2 and TRAF4 specific siRNA into MCF7 together with TNFa treatments. As shown in Figure 7D, 7E, knocking down of TRAF2 or TRAF4 remarkably reduced TNFa-induced upregulation of p-p70s6k and p-S6. These results suggested that both TRAF2 and TRAF4 are essential in TNFa-induced activation of p70s6k/S6 pathway.

In order to confirm whether TRAF2 lies upstream of TRAF4 in TNFa-induced p70s6k pathway activation, we detected the localization of TRAF2 and TRAF4 after treatment with TNFa by immunofluorescence staining. As shown in Figure 7F, we found translocation of TRAF2 to the cytosolic side near the cell membrane of MCF7, and the co-localization of TRAF4 and TRAF2 was obviously detected in the cytosolic side near the cell membrane. In order to further demonstrate the precise mechanism that TRAF2 and TRAF4 participated TNFa-induced p70s6k pathway activation, we detected the interaction between mTOR, p70s6k, TRAF4 and TRAF2 after TNFa treatment. As shown in Figure 7G, TNFa stimulation increased p70s6k interaction with mTOR. We also found that more TRAF4 proteins were interacting with p70s6k and TRAF2 after TNFa incubation. We therefore concluded that after TNFa stinulation, TNFa interacted with TNFR, and then TRAF2 accumulated to the cytosolic region of TNFR, then TRAF2 interacted with TRAF4 and TRAF4 interacted with p70s6k, next enhancing the p70s6k pathway activation.

### 6. TRAF4 promotes the proliferation of breast cancer cells mostly through p70s6k/S6 signaling

**Figure 8:**
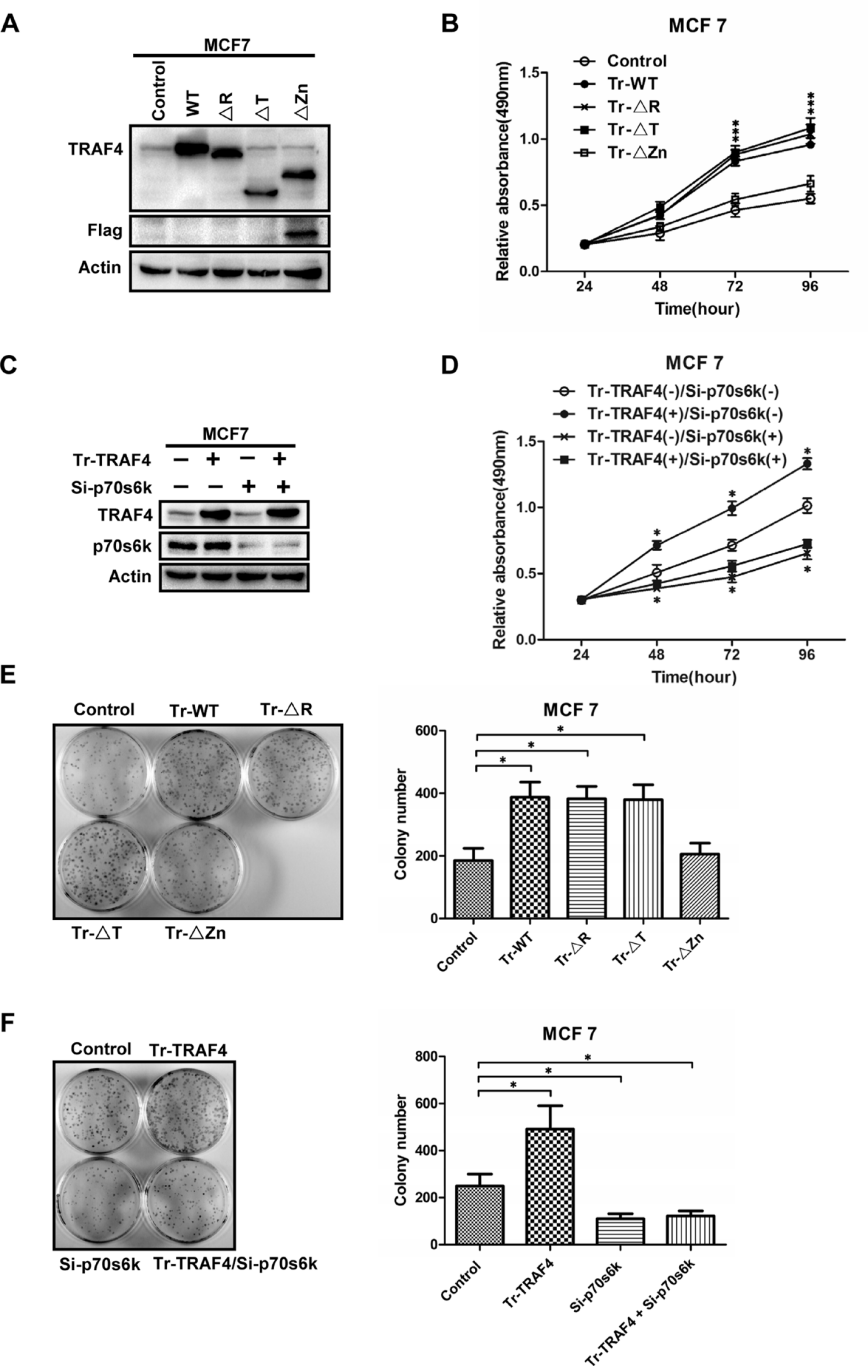
TRAF4 promotes the proliferation of breast cancer cell mostly through mTOR/p70s6k/S6 signaling **(A)** Western blot of wild type and TRAF4 mutants transfection efficiency in MCF7 cells. **(B, E)** TRAF4 promotes the proliferation of MCF7 and Zinc fingers domain was critical for it. MTT assay and colony formation assay were performed to analysis the proliferation of MCF7 after it was transfected with WT-TRAF4, ΔR, ΔT and ΔZn mutants vectors. Overexpression of WT-TRAF4, ΔR, and ΔT mutants all significantly promoted the proliferation of MCF7, but overexpression of ΔZn mutant had no obviously or just a little effection on proliferation of MCF7. **(C)** Western blot of TRAF4 transfection and p70s6k depletion efficiency in MCF7 cells. **(D, F)** TRAF4 promotes the proliferation of breast cancer cell mostly through p70s6k/S6 signaling in MCF7 cells. MTT assay and colony formation assay were performed to analysis the proliferation of MCF7. Knockdown p70s6k significantly but not absolutly inhibited the promoting effect of TRAF4 on proliferation of MCF7. Data represent mean ± SD (*n* = 3). (*p < 0.05).

TRAF4 could promote cell migration through modulating the tight junctions between cells [28]. It also drives breast cancer metastasis through promoting TGF-b receptor signaling [29]. In this study, we validated that TRAF4 could promote the activation of p70s6k/S6 pathway which was involved in control of cell growth, survival and cell cycle progression. So, TRAF4 may promote the proliferation of breast cancer cell through p70s6k/S6 signaling pathway. To confirm our hypothesis, we transfected the expression vectors for full length TRAF4, ΔR, ΔZn and ΔTRAF mutants into MCF7. Western blot was performed to evaluate the transfection efficiency (Figure 8A). MTT assay and Colony formation assay were performed to analyze the proliferation of MCF7 cells. The results indicated that wild type TRAF4, ΔR and ΔT mutants all significantly enhanced the growth of MCF7 when compared to the control, while ΔZn mutant had no significant effect on the growth of MCF7 when compared to the control (Figure 8B and 8E). The above results suggest that TRAF4 promoted the proliferation of MCF7 cells and Zinc fingers domain of TRAF4 was required for its promoting function on MCF7 proliferation.

To confirm whether TRAF4 promoted the proliferation of MCF7 through its upregulation on the p70s6k/S6 pathway, we transfected TRAF4 expression vector into MCF7 together with p70s6k-specific siRNA to knockdown p70s6k expression. Western blot was performed to test the transfection and RNA interference efficiency of TRAF4 and p70s6k respectively (Figure 8C). MTT assay and Colony formation assay were performed to analyze the proliferation of MCF7 cells. The results showed that knockdown of p70s6k strongly reduced the promoting effect of TRAF4 on growth of MCF7 cells (Figure 8D, 8F). These results suggest that p70s6k played a key role in the TRAF4-induced enhancement of proliferation of MCF7 cells.

## DISCUSSION

TRAF4, the unique member of the TRAF family, has been studied for several decades since it was first identified in breast cancer. There have been inconsistent conclusions about the cellular function of TRAF4 according to its different localization. In this study, we primarily studied the function of the cytoplasm localization of TRAF4 *in vivo* and *in vitro*.

Basing on the interaction of TRAF4 and p70s6k [15], we further analyzed their relationship with immunohistochemistry and western blot in breast cancer tissues (*in vivo*). Our results suggested that TRAF4 cytoplasm expression was significantly associated with poor prognosis. Interestingly, it has been reported recently that high TRAF4 nuclear expression is also correlated with poor survival in breast cancer patients [6]. These seemingly disparate results show that both cytoplasmic and nuclear TRAF4 expressions played an important role in progression of breast cancer through different pathways. In summary, the overexpression of TRAF4 indicates poor survival of breast cancer patients. In the future, TRAF4 may become one molecular marker for therapy of breast cancer.

Unlike the RING finger domain and TRAF domain. Zinc fingers domain has not been studied very well. Here, we elucidated the key role of Zinc fingers domain in cellular function of TRAF4. We also confirmed that TRAF4 interacts with p70s6k through its Zinc fingers domain. Moreover, we demonstrated that the promoting function of TRAF4 on the p70s6k/S6 pathway was depend on mTOR activity, because Rapamycin, the specific inhibitor of mTOR, could significantly suppress the promoting function of TRAF4 on p70s6k signaling pathway. Furthermore, the interaction of TRAF4 and p70s6k was also significantly inhibited by Rapamycin. Therefore, we prefer to consider TRAF4 as a sensitizer in the mTOR/p70s6k/S6 pathway. As we know, the activation of p70s6k is tightly regulated and it includes the conformation change and several phosphorylation sites of p70s6k [9]. In the study, we have found that TRAF4 overexpression could make more p70s6k binding mTOR which indicates more p70s6k was phosphorylated by mTOR. How the interaction of TRAF4 and p70s6k enhances the sensitivity of mTOR to its substrate p70s6k has yet to be studied. We propose the hypothesis that TRAF4 changed the conformation of p70s6k through interacting with it. Following the conformation change, the phosphorylation site is exposed to the outside and then it would be easier to be phosphorylate and activate. However, this hypothesis needs to be further studied.

TRAF2 and TRAF4 are all TRAFs family members and we have found TRAF2 could interact with TRAF4 [16]. In this study, we confirmed the interaction between TRAF2 and TRAF4 and further identified the TRAF domain was the exact TRAF2-interacting region in TRAF4. It is well known that the TRAF domain is highly conserved in TRAF family members; it can mediate TRAF homodimerization, trimerization, or heterodimerization with other TRAFs [27]. The interaction between TRAF2 and TRAF4 may be analogous to the heterodimerization between other TRAF family members. Based on the regulation of TRAF2 on TRAF4, we examined whether TRAF2 affected p70s6k/S6 pathway through regulation of TRAF4 expression. As we expected, TRAF2 could indeed promote p70s6k/S6 pathway activation through upregulating cytoplasmic TRAF4 expression. It also indirectly confirmed the important role of cytoplasmic TRAF4 on promoting p70s6k/S6 pathway activation.

TRAF4, as a factor associated with TNF receptor, made us naturally link it with the TNFa induced signaling pathway. Based on that, TNFa can induce p-S6 upregulation with TRAF4 involvement [15], but the molecular mechanism has not been studied clearly. Specifically, there is one scaffold protein linking TNF receptors and the p70s6k/S6 pathway that has yet to be identified. Our results in the study suggest that TRAF2 is the adaptor that mediated the TNFa induced p70s6k activation through accumulation on TNF receptors, which also depends on TRAF4 participation.

The p70s6k/S6 pathway was involved in several cell biology behaviors, such as cell survival and proliferation, according to the promotional effect of TRAF4 on p70s6k/S6 pathway. Here, we demonstrated that TRAF4 promotes the proliferation of breast cancer cell MCF7 predominantly through the p70s6k/S6 pathway, with the Zinc fingers domain playing a key role.

**Figure 9:**
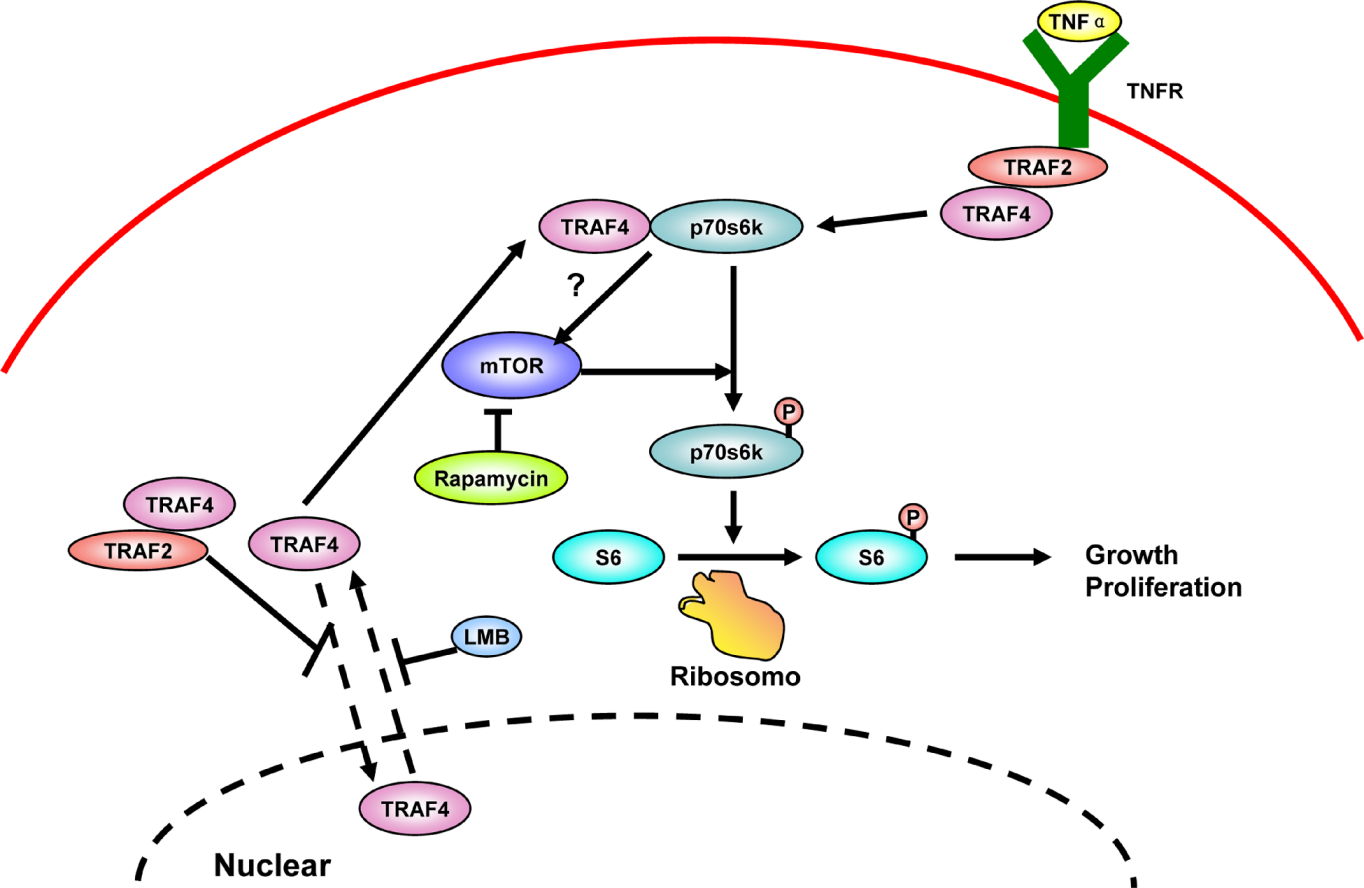
Schematic representation of the proposed mechanism, linking TRAF4 to activation of p70s6k/S6 signaling pathway in the control of cell proliferation

In summary, we demonstrated that cytoplasmic TRAF4 has pivotal roles in regulating the activation of p70s6k signaling in breast cancer. Furthermore, we also identified TRAF2 as the adaptor that mediated the TNFa induced p70s6k activation, with the participation of TRAF4 (Figure 9). These findings greatly advanced our understanding of TRAF4's role in oncogenesis, representing a promising field of future investigation for cancer research and anti-cancer therapeutics, especially in breast cancer.

## METHODS

### Ethics Statement

The study was conducted with the approval of the Ethics Review Committee at the First Affiliated Hospital of China Medical University. Fresh samples and the paraffin embedded breast cancer samples were obtained from patients at First Affiliated Hospital of China Medical University. Informed consent had been obtained. Two pathologists independently identified the pathologic diagnoses based on World Health Organization guidelines.

### Immunohistochemistry

The paraffin embedded samples were cut serially into 4 μm thick sections. The tissue sections were deparaffinized in xylene, rehydrated in graded alcohol series and boiled in 0.01 M citrate buffer (pH 6.0) for 2 minutes in an autoclave. Endogenous peroxidase activity was blocked using hydrogen peroxide (0.3%), which was followed by incubation with normal goat serum to reduce non-specific binding. The tissue sections were then incubated with TRAF4 antibody (1:100 dilution, Santa Cruz Biotechnology, H72) and p-p70s6k(T389) antibody (1:100 dilution, Cell signaling technology, 9206) at 4°C overnight. PBS was used as a negative control. Biotinylated goat anti-mouse/rabbit serum IgG (ready-to-use, cat. KIT-9922, MaiXin Bio) was used as the secondary antibody. After washing, the sections were incubated with horseradish peroxidase-conjugated streptavidin–biotin, followed by 3, 39-diaminobenzidine tetrahydrochloride to develop the peroxidase reaction. Counterstaining of the sections was performed with hematoxylin, and then the sections were dehydrated in ethanol before mounting. Two independent investigators examined all the tumor slides randomly. Five random views were examined per slide, and 100 cells were observed per high magnification field (400 ×). The TRAF4 and p-p70S6K expression levels were evaluated based on the percentage of positive cells (PP) and staining intensity (SI) within the whole tissue section. TRAF4 cytoplasmic expression was scored semiquantitatively using the immunoreactive score (IRS), which was calculated as follows: IRS = PP × SI. PP: 0 = no staining; 1 = 1–25%; 2 = 26–50%; 3 = 51–75%; and 4 = 76–100%. SI: 0 = no staining; 1 = weak; 2 = moderate; 3 = strong staining. When IRS < 3, the case was defined as negative. When IRS >= 3, the case was defined as positive. During the positive cases, when 3 =< IRS < 6, the case was defined as low expression; when 6 =< IRS <= 12, the case was defined as high expression. TRAF4 nuclear staining was scored according to PP only. When PP >= 2, the case was defined as positive. The p-p70S6k staining was evaluated by an H score [30, 31]. When H scores > 50, the case was considered positive.

### Cell lines and reagents

The human breast cancer cell lines, MCF-7, MDA-MB-231, and one human non-malignant breast epithelial cell line MCF10A were obtained from the American Type Culture Collection (ATCC; Manassas, VA, USA). MCF10A cells were cultured in DMEM/F12 (1:1) supplemented with 5% equine serum, 10 μg/ml insulin and 20 ng/ml EGF. MDA-MB-231 cells were cultured in L15 supplemented with 10% FBS. MCF7 cells were routinely cultured in DMEM supplemented with 10% FBS. All of the cells were cultured at 37°C with 5% CO2 in a humidified incubator. Antibodies specific to p-p70S6K (T389, #9206), p70s6k (#2708), S6 (#2317), phosphor-S6 (Ser235/Ser236, #4856) were all obtained from Cell Signaling Technology. Antibodies to TRAF4 (sc-10776) and LaminB1 (sc-30264) were obtained from Santa Cruz Biotechnology. Antibody to TRAF2 (#558890) was obtained from BD Pharmingen. Antibodies to mTOR, Tubulin, Flag and Actin were obtained from Beyotime (China). Rapamycin (#9904, CST). Human TNFa (#8902, CST). Leptomycin B (LMB, sigma-aldrich, USA, final concentration, 50 nM 20 h) was used to block the nuclear export of TRAF4.

### Plasmid and transfection

Plasmid for full length TRAF4 (wild type, WT), Ring domain deletion mutant (ΔR), TRAF domain deletion mutant (ΔT), and TRAF2 were obtained from Addgene (Cambridge, USA). The plasmid for Zinc fingers domain deletion mutant (ΔZn) was provided by Dr. Bert W. O'Malley. The TRAF4 siRNA sequence was purchased from Santa Cruz Biotechnology. p70s6k siRNA sequence sense strand: 5′-GGACAUGGCAGGAGUGUUUtt-3′, antisense strand: 5′-AAACACUCCUGCCAUGUCCtc-3′ [32]. TRAF2 siRNA sequence sense strand: 5′-GGACCAAGACAAGAUUGAATT-3′, antisense strand: 5′-UUCAAUCUUGUCUUG GUCCTT-3′ [33]. Cells were transfected with plasmids using the Attractene Transfection Reagent or siRNA using HiPerFect Transfection Reagent (Qiagen, Hilden, Germany) according to the manufacturers' protocols. The empty plasmid and scrambled sequences were used as control.

### Cellular fractionation

Nuclear and cytoplasmic fractions were isolated by using the NE-PER Nuclear and Cytoplasmic Extraction kit (Thermo scientific, Barrington, IL, USA) according to the manufacturer's instructions.

### Immunoprecipitation (IP)

It was carried out by using whole cell lysates (200 μg protein) with 1–2 μg of antibody, and 20 μl of protein A/G agarose (Beyotime, china). The cell lysates were precleared with 20 μl (50% slurry) agarose A/G beads by rocking for 1 h at 4°C. Beads were removed and appropriate antibodies were added to the precleared lysates overnight at 4°C. 20 μl (50% slurry) agarose A/G beads were added to the lysates by rocking for 4 h at 4°C. The immune complex was washed in cell lysis buffer before analysis by immunoblotting.

### Immunofluorescence staining

The Cells were seeded in glass coverslips and fixed with 4% paraformaldehyde in phosphate-buffered saline for 20 min and then permeabilized with 0.2% Triton X-100 for 15 min at room temperature. After washing with phosphate-buffered saline, cells were blocked with normal goat serum for 1 h at room temperature, and then incubated with primary antibody overnight at 4°C. Cells were then incubated with the second antibodies (Dylight488 labeled goat anti-rabbit, Dyligh594 labeled goat anti-rabbit, Dyligh594 labeled goat anti-mouse) and mounted with DAPI. Images were acquired by using a Radiance 2000 laser scanning confocal microscope (Carl Zeiss, Thornwood, NY, USA).

### Western blot analysis

Cells were disrupted on ice in cell lysis buffer for Western and IP containing 20 mM Tris–HCl pH 7.5, 150 mM NaCl, 10 mM EDTA, 1% Triton X-100, 40 mM β-glycerophosphate, 10 mM sodium pyrophosphate, and 10% of a protease inhibitor cocktail (SIGMA). Protein concentration was quantified by using the Bradford reagent and Equal amounts (50~100 μg) of the total protein was used for protein immunodetection analysis. The lysates were separated by SDS-PAGE and transferred to PVDF membrane (millipore). Membranes were blocked for 2 h at room temperature with 5% non-fat milk or 5% BSA in Tween TBS and incubated with antibodies against TRAF4 (1:200), TRAF2 (1:500), p70s6k (1:1000), p-p70S6k (1:1000), S6 (1:1000), p-S6 (1:1000), mTOR (1:500), Tubulin (1:1000), laminB1 (1:500), Flag (1:1000), Actin (1:1000) at 4°C for overnight. Secondary HRP-conjugated antibody (1:1000) was included for 2 h at room temperature. The protein bands were visualized using enhanced chemiluminescence (ECL, Thermo Fisher Scientific, Waltham, MA, USA) and quantified using BioImaging Systems (UVP, Upland, CA, USA).

### Cell proliferation analysis

Cell proliferation was determined by MTT (3-(4, 5-Dimethylthiazol-2-yl)-2, 5-diphenyltetrazolium bromide)assay. The absorbance was evaluated after the MTT treatment and was measured at 490 nm using a microplate reader (Model 550, Bio-Rad, Hercules, CA, USA). A blank with dimethyl sulfoxide (DMSO) alone was measured and the value was subtracted from all the absorbance for cell culture specimens.

### Colony formation assay

After cells were treated with the study plasmid or siRNA for 24 h, 5–10 × 10^2^ cells were plated in 35 mm dish and incubated for 10–12 days until visible colonies formed. The colonies were then fixed with methanol, stained with hematoxylin and scored using a microscope after the plates were air dried.

### Statistical analysis

SPSS 17.0 was used for all statistical analysis. The Pearson chi-square test and Fisher's exact test were used to analyze the relationship of TRAF4 and p-p70s6k expression. The Kaplan-Meier method was used for survival curves and log-rank test was used for comparison. The Student's *t*-test was used to analyze the statistical significance. Experiments were peformed three times. The data was expressed as mean ± SD for cell proliferation experiments. *p* < 0.05 was considered statistically significant.
